# Are We Biologically Safe with Snow Precipitation? A Case Study in Beijing

**DOI:** 10.1371/journal.pone.0065249

**Published:** 2013-06-06

**Authors:** Fangxia Shen, Maosheng Yao

**Affiliations:** State Key Joint Laboratory of Environmental Simulation and Pollution Control, College of Environmental Sciences and Engineering, Peking University, Beijing, China; National Center for Biotechnology Information (NCBI), United States of America

## Abstract

In this study, the bacterial and fungal abundances, diversities, conductance levels as well as total organic carbon (TOC) were investigated in the snow samples collected from five different snow occurrences in Beijing between January and March, 2010. The collected snow samples were melted and cultured at three different temperatures (4, 26 and 37°C). The culturable bacterial concentrations were manually counted and the resulting colony forming units (CFUs) at 26°C were further studied using V3 region of 16 S rRNA gene-targeted polymerase chain reaction -denaturing gradient gel electrophoresis (PCR-DGGE). The clone library was constructed after the liquid culturing of snow samples at 26°C. And microscopic method was employed to investigate the fungal diversity in the samples. In addition, outdoor air samples were also collected using mixed cellulose ester (MCE) filters and compared with snow samples with respect to described characteristics. The results revealed that snow samples had bacterial concentrations as much as 16000 CFU/ml for those cultured at 26°C, and the conductance levels ranged from 5.6×10^−6^ to 2.4×10^−5^ S. PCR-DGGE, sequencing and microscopic analysis revealed remarkable bacterial and fungal diversity differences between the snow samples and the outdoor air samples. In addition, DGGE banding profiles for the snow samples collected were also shown distinctly different from one another. Absent from the outdoor air, certain human, plant, and insect fungal pathogens were found in the snow samples. By calculation, culturable bacteria accounted for an average of 3.38% (±1.96%) of TOC for the snow samples, and 0.01% for that of outdoor air samples. The results here suggest that snow precipitations are important sources of fungal pathogens and ice nucleators, thus could affect local climate, human health and agriculture security.

## Introduction

Particles of biological origins are ubiquitous in the atmosphere [1]. Studies showed that bioaerosols, such as bacteria and fungi, accounted for approximately 24% of the total particles in the atmosphere [2], [3]. In addition to their direct health effects [4]–[7], numerous studies indicated that atmospheric bioaerosols also play an important role in the atmospheric precipitation processes, such as rain and snow [8]. Many bacterial and fungal spores are found to be acting as efficient ice nuclei, and to initiate the freezing at temperatures as high as −2°C [9], [10]. Accordingly, cloud water, fog, and snow all could serve as a microbial habitat [11]–[15]. In a study, about 25% of the total insoluble particles in cloud waters were shown to be biological ones [14]. In snow samples from Austrian Alps, the concentrations of bacteria and fungi were found to be 3.1×10^3^ cells ml^−1^ and 6.2×10^2^ spores ml^−1^, which corresponds to 0.015% of total carbon (TC), and corresponding to 1.8% of TC, respectively [16]. At an altitude of 48 to 77 km in the stratosphere, cultivable bacteria and fungi have also been observed [17]. In a recent review article, it was pointed out that the atmosphere is a habitat for microorganisms, not just a conduit for terrestrial and aquatic life [18], [19]. Thus, snow precipitation could be an important source for local microbial diversity and might have direct impact on local environmental health.

There have been increasing evidences that the biological agents could be transported globally [20]–[27]. Morris and co-workers (2007) showed that pathogens, such as *Pseudomonas syringae* (a plant pathogen) might involve in long-range transboundary atmospheric transport [19], and they could catalyze the ice formation [1]. The transported pathogens such as *P. syringae* thus can precipitate via snowfalls to the ground, possibly leading to the local agriculture injuries. Microorganisms attached to Saharan dust were found to be transported across the Atlantic Ocean [28]. The interhemispheric transport of viable fungi and bacteria via soil dust from Africa to the Caribbean was also reported [29]. In a recent study, the air samples from the elevation of 238 m were shown to have similar airborne microbial community to those obtained from the ground possibly due to the atmospheric mixing [30]. These evidences show that the atmospheric movement affected the bioaerosol dynamics. In another study, it was found that a large portion of the microbes detected in the atmosphere at respiratory particle sizes (<3.3 µm) appeared to be phylogenetic neighbors to human pathogens [31], implying that they could pose negative impacts on human health, agricultural and ecosystem health.

For the microbial pollutant transport, the snow precipitation as aforementioned might play an important role. According to Christner and co-workers (2008), DNA-containing cells between 0.3 and 15 µm in diameter had a concentration of 1.5× 10^4^ to 5.4×10^6^ cells per liter in their snow samples collected [1]. In another study, rich microbial classes including 19 different ones were retrieved from snow samples collected in Arctic [32]. Liu and co-workers (2007) had found that bacterial abundance tended to increase with the altitude, but showed no obvious correlation with ion concentrations [33]. In another study, it was shown that the snow bacterial community structures in different seasons were diverse, including not only common species but season-specific species [34]. These transported snow-borne pollutants including diverse forms of bacteria, fungi, and plant pathogens could further settle into the local environments through atmospheric mixing or atmospheric precipitations such as snow or rain, which could cause relevant health effects to humans and injuries to the agriculture. However, fewer studies were conducted to investigate such a possibility. In addition, the dynamic microbial structures of different snow samples are less reported.

In 2010, consecutive snow precipitations occurred in Beijing between January and March. Taking this opportunity, we carried out this work to investigate if snow precipitations could represent an important source of microbial species and infections. In addition, we also investigated if the snow samples differed from one another with respect to biological contents. Besides, we also measured the conductance and TOC (bacteria are important contributor to both) for snow samples and outdoor air samples. Bacterial diversity was studied by analyzing the colony forming units using 16 S rRNA gene-targeted PCR-DGGE and constructing clone library after sequencing, and fungal genera were identified through optical microscopic observation. All samples collected were cultured simultaneously at three different temperatures (4, 26, and 37°C).

## Materials and Methods

### Snow Collection and Outdoor Air Sampling

There were five consecutive snow precipitations in Beijing on Jan 1, Feb 7, Mar 1, Mar 8, Mar 14, 2010. In addition, snow samples were also collected on Feb 27, 2011. For each snow occurrence on a different date, fresh snow samples (on the top of the snow layer) in the middle of or right after the snow precipitation were collected from the top layer into 50 ml tubes from five different locations (to mimimize the variations from the snow samples from each occurrence) within the campus of Peking University, located at the 4th Ring and 5th Ring of Beijing city. For each snow occurrence, the snow samples collected from five different places within the campus were pooled together for analysis. Considering upper atmospheric temperature, the snow samples were stored at −70°C in a freezer (Thermo Fisher Scientific, Marietta, OH) before analysis. In addition, for microbial diversity analysis the outdoor air samples within Peking University campus were also collected using an Aerosol Button Sampler (SKC) with mixed cellulose ester (MCE) (SKC Inc., Eighty Four, PA) filters at a flow rate of 13.5 L/min for 40 min on a date (Feb 20, 2010) between these five snow occurrencies. High flow rate rather than its standard 4 L/min was used to collect enough air samples for limiting dessication effects of longer sampling time. For culturable concentration study, the filter sample was placed in a corning tube (Corning Inc., Acton, MA) filled with 4 ml DI water (Millipore Co., Billerica, MA), and then extracted using a sonicator (Kunshan Ultrasonic Instruments Co. Ltd., Shanghai) for about 20 min. The resulting suspension was stored at −4°C for subsequent experiments. For denaturing gradient gel electrophoresis (DGGE study), three filter samples collected at 10 L/min for 20 min were also used. Blank agar plates or DI water were used as negative controls for culturing and PCR-DGGE analysis.

### Sample Culturing and PCR-DGGE

#### Sample culturing

Immediately before analysis, the snow samples were melted at room temperature. Then, 2 ml of the melted snow was taken out from the snow tube and diluted 10 times using DI water. For the snow samples (pooled together) collected on each occurrence on a particular date, 80 µl of the original suspesion (diluted 10 times) was pipetted onto on a Trypticase Soy Agar (TSA) (Becton, Dickson and Company, Sparks, MD) plate for bacteria culturing. Likewise, 80 µl of the original suspesion without dilution was pipetted onto a Malt Extract Agar (Becton, Dickson and Company, Sparks, MD) plate for fungal culturing at 26°C. For bacteria, the snow samples were grown at three different temperatures: 4°C, 26°C and 37°C. Bacterial and fungal aerosols are often cultured at 26°C and 37°C, while use of 4°C could readily activate those bioaerosols acting as ice nucleators. For each sample, three replicates were perfromed. The colony forming units (CFUs) were manually counted and the bacterial concentration was calculated as CFU/ml of melted snow suspenison. For culturable concentrations, 100 µl of filter extraction liquid was plated onto an agar plate with three replicates for the outdoor air sample. For DGGE study, the fiter samples were directly placed on agar plates for culturing at 26°C.

#### Bacterial diversity analysis

For bacterial diversity analysis, the CFUs from the agar plates both for the outdoor air and snow samples were washed off using DI water to a total volume of 5 ml. Then, 1 ml of the bacterial suspension was used for DNA extraction by a bacterial DNA extraction kit (Tiangen, Beijing) following the manufacturer’s instruction. The extracted DNA samples were further resuspended into 50 µl DI water. The V3 region of the 16 S rRNA gene was amplified using primers P2 (5'-ATTACCGCGGCTGCTGG-3') and P3 (5'-GCclamp-CCTACGGGAGGCAGCAG-3') as used by Muyzer et al. (1993) [35]. PCR reaction mixture (total volume 50 µl) included 5 µl DNA template, 2 µl primer P2 (10 µM), 2 µl primer P3 (10 µM), 25 µl 2×Master Mix (10X Taq Buffer, dNTP Mixture, Taq (2.5 U/µl) (Tiangen, Beijing) and 16 µl DI H_2_O. The cycle conditions were set as : 94°C for 3 min, 30 cycles of [94°C for 30 sec, 55°C for 30 sec and 72°C for 1 min], and 72°C for 10 min. DI water was used as the negative control in the PCR experiments. The DGGE analysis of PCR amplicons was performed using a Bio-Rad DcodeTM mutation detection system (Bio-Rad, Hercules, CA) according to the manufacturer’s instructions. In details, approximately 20 µl PCR product was transferred to each well of 8% polyacrylamide (acrylamide/bisacrylamide: 35.7∶0.8) gels which contain a linear 30–65% gradient of urea and formamide increasing in the direction of electrophoresis. The electrophoresis was conducted in 1× TAE buffer for 540 min at a constant voltage of 100 V at 60°C. DNA bands in gels were stained by GelRed solution (10000 x diluted with DI water) (Biotium, Hayward, CA) and photographed (Molecular Imager Gel DocTM XR system, Bio-Rad, Hercules, CA) under ultraviolet lamp at the wavelength of 302 nm. Data were recorded and analyzed with Quantity One software (Bio-Rad). Here, the bands were marked by an arrow, and all lanes were compared with each other. The DGGE bands for the snow samples and the outdoor air sample were further compared using dendrograms obtained using the BioRad built-in software functions “band detection” and “unweighted pair group method with arithmetic mean (UPGMA)” clustering, and the similarity of the culturable microbial aerosol diversities was analyzed using the scale bar produced by the methods.

For method comparison and optimization, the bacteria diversity in the snow samples and the outdoor air samples was also studied using another two different culturing steps: liquid culturing of snow samples and liquid-plate culturing (first cultured using liquid broth, then cultured again using agar plate). After the culturing, the same procedure described for agar plate culturing was adopted in applying the PCR-DGGE analysis to the CFUs. In addition, the diversity was studied directly by PCR-DGGE procedure without the culturing, in which 1 ml of the melted snow sample was used for direct DNA extraction.

Besides, we also constructed clone libraries of V3 region of 16S rRNA gene followed by sequence analyses. The enrichment of melted snow samples was performed using liquid culturing method. After three days’ culturing, 1 ml of each cultured snow sample were extracted for DNA. The V3 region of 16 S rRNA gene of all the extracted DNA were amplified as mentioned above. The PCR products were purified using an agarose gel DNA purification kit (Beijing Transgen Biotech Co., Ltd, Beijing), ligated into a pEASY-T1 simple cloning vector, and then transformed into Trans1-T1 phage resistant chemically competent cell following the manufacturer’s instructions (Beijing Transgen Biotech Co., Ltd). The presence and size of inserts were checked by blue-white selection method followed by PCR and gel electrophoresis. These amplicons were further sequenced using the M13 primers (Majorbio Bio-Pharm Technology Co., Ltd., Shanghai, China). All sequences were aligned using the MEGA 5.0 software [36]. Then, the aligned sequences were clustered to OTUs (operational taxonomic units) using Mothur [37]. Sequences with similarity scores greater than or equal to 97% were clustered into an operational taxonomic unit. For comparison with known sequences, the representative sequence of each OTU was used as query sequence using the Basic Local Alignment Search Tool (BLAST) to find the most closely relative sequence. The representative sequences from clone libraries and the reference sequences were aligned using ClustalW, followed by the phylogenetic analysis using MEGA 5.0 [36]. The phylogenetic tree was constructed using the neighbor joining algorithm.

### Fungal Diversity Analysis

For culturable fungal aerosols, microscopy method was used for fungal genus identification in both snow and outdoor air samples. Such a method is widely used in other studies [38]–[40]. Fungal colonies were first washed off from the agar plates using 10 ml DI water (Millipore), and about 5 µL of fungal suspension was pipetted onto the glass slide. 1000 times magnification was achieved using the immersion oil for Olympus CX 41 to capture the images of the fungi that were recognized using a Nikon camera. The fungal genera were identified through visual comparison of the images taken in this study with those already known fungal morphologies according to a reference book [41].

### Snow Conductance and TOC Measurments

In this study, the conductance levels in snow samples were also measured using a lock-in amplifier (LI5640, NF Corporation, Yokohama, Japan). Before the sample analysis, different frequencies of 1 kHz to 100 kHz were automatically tested using a LabView program developed in our lab. For each snow sample, 20 µl of melted snow sample was used for the measuremnt, and total 500 measurements were made (1 measurement per 300 millisecond). The optimal frequency from those tested at which the conductance level remains relatively stable was selected for the final sample conductance analysis. Using the optimal frequency, the average conductance levels of the mixture from five different snow samples collected on each occurrence on a particular date were measured. In addition, the total organic carbon concentations for all samples (an outdoor air sample after filter extraction and the mixtures of five snow samples for each date) were also measured using a TOC analyzer (Analytik Jena AG, Jena, Germany). Here, we also calculated the bacterial contribution to the total oragnic carbon using a conversion factor of 17 pg carbon per bacterium that was established in a previous study [42].

### Statistical Analysis

The concentration levels of culturable bacteria of snow samples and outdoor air samples under different culturing temperatures, and the conductance levels of all snow samples were analyzed using paired t-test and Analysis of Variance (ANOVA). These data are normally distributed. A p-value of less than 0.05 indicated a statistically significant difference at a confidence level of 95%.

## Results

### Bacteria in the Snow Samples

**Figure 1:**
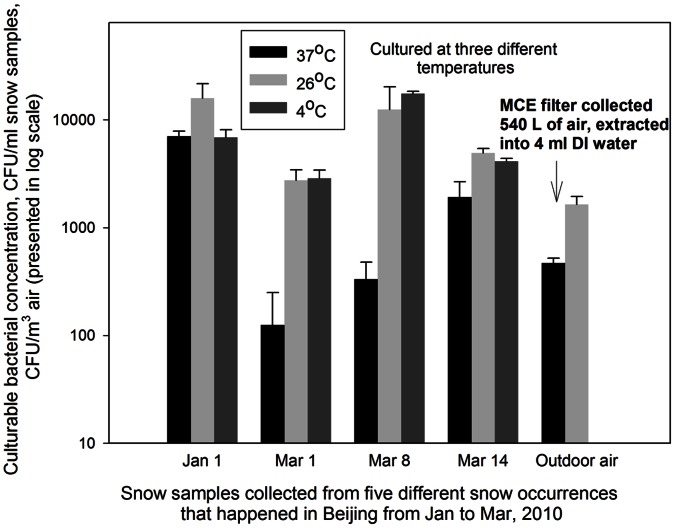
Culturable bacterial concentrations when cultured at three different temperatures (4, 26, and 37°C) in different snow samples and also in an outdoor air sample collected in Beijing from January to March, 2010. Collected snow samples were melted at room temperature immediately before the analysis; snow data represent averages of five snow samples collected for each snow occurrence on a particular date, and error bars represent standard deviation of three replicates; blank agar plates were used as negative controls.

This study investigated the biological contents, conductance levels as well as total organic carbons in snow samples collected from five snow occurrences that happened during January-March 2010 in Beijing, and the relevant results were compared among them as well as with those in outdoor air samples. Fig. 1 shows the culturable bacterial concentrations in the snow samples and outdoor air samples obtained by culturing at three different temperatures: 4, 26 and 37°C. As observed in Fig. 1, in most cases the culturing of snow samples at 26°C resulted in the highest culturable bacterial concentrations, followed by culturing at 4 and 37°C. For two snow samples, the culturing at 4°C produced slightly higher concentrations than the culturing at 26°C. For all samples, the culturing at 37°C resulted in lowest concentration levels. Compared to the snow samples, outdoor air samples cultured at 4°C for 2 days produced very few colony forming units (below 10) under similar conditions. The culturable bacterial concentration levels for the snow samples here were found to range approximately from 2750 to 16000 CFU/ml of melted snow at 26°C, while those for the outdoor air sample were about 1600 CFU/m^3^. These data suggested that bacteria were abundant in the snow samples collected, and bacteria in the snow samples can be more active at lower temperatures than those (likely in the state of viable but not culturable) in outdoor air samples, implying they might play possible roles in ice nucleation and cloud formation. ANOVA tests indicated there were statistically significant differences in the bacterial concentrations obtained for five snow samples for different occurrences at different incubation temperatures (p-values = 0.001–0.028).

In this work, the bacterial concentration levels for the snow samples collected in Beijing were found in similar magnitudes with those in snow samples reported in previous studies. In a previous study, it was found that the bacterial concentration in snow samples collected from Mt. Everest ranged from 5.7×10^3^ to 2.3×10^4^ cells/ml [34]. In another study, the DNA-containing particles of 0.3–15 µm in diameter from the Antarctic snowfalls were found between 1.5×10^4^ and 5.4×10^6^ cells/L [1]. Similar levels of bacterial concentration were found in snow samples collected from the Austrian Alps [16]. Such bacterial abundances were also observed in snow samples in other studies [32], [43]. These evidences suggest that snow is one of the bacterial favorable habitats in the atmosphere, where the bacterial cells might have served as the ice nucleators (INs). There is such a growing body of evidences that biological aerosols could participate in the ice nucleation and the cloud formation processes [1], [44]–[45], [47], [48]. This was further strengthened by a recent study in which it was shown that 69 to 100% of the INs larger than 0.2 µm that were active at temperatures warmer than −7°C were biological particles, and a substantial fraction were bacteria [1]. Here, we also observed the differences in the culturable fraction of the bacteria in the snow samples collected on different dates when cultured under similar conditions, and such differences were statistically significant (p-values <0.05). In agreement with previous studies, the results shown in Fig. 1 also exhibited the bacterial abundance in the snow samples collected. The results also suggest that bacteria in snow samples tend to be temperature tolerant for their growth.

### Bacterial Diversity in the Snow Samples

**Figure 2:**
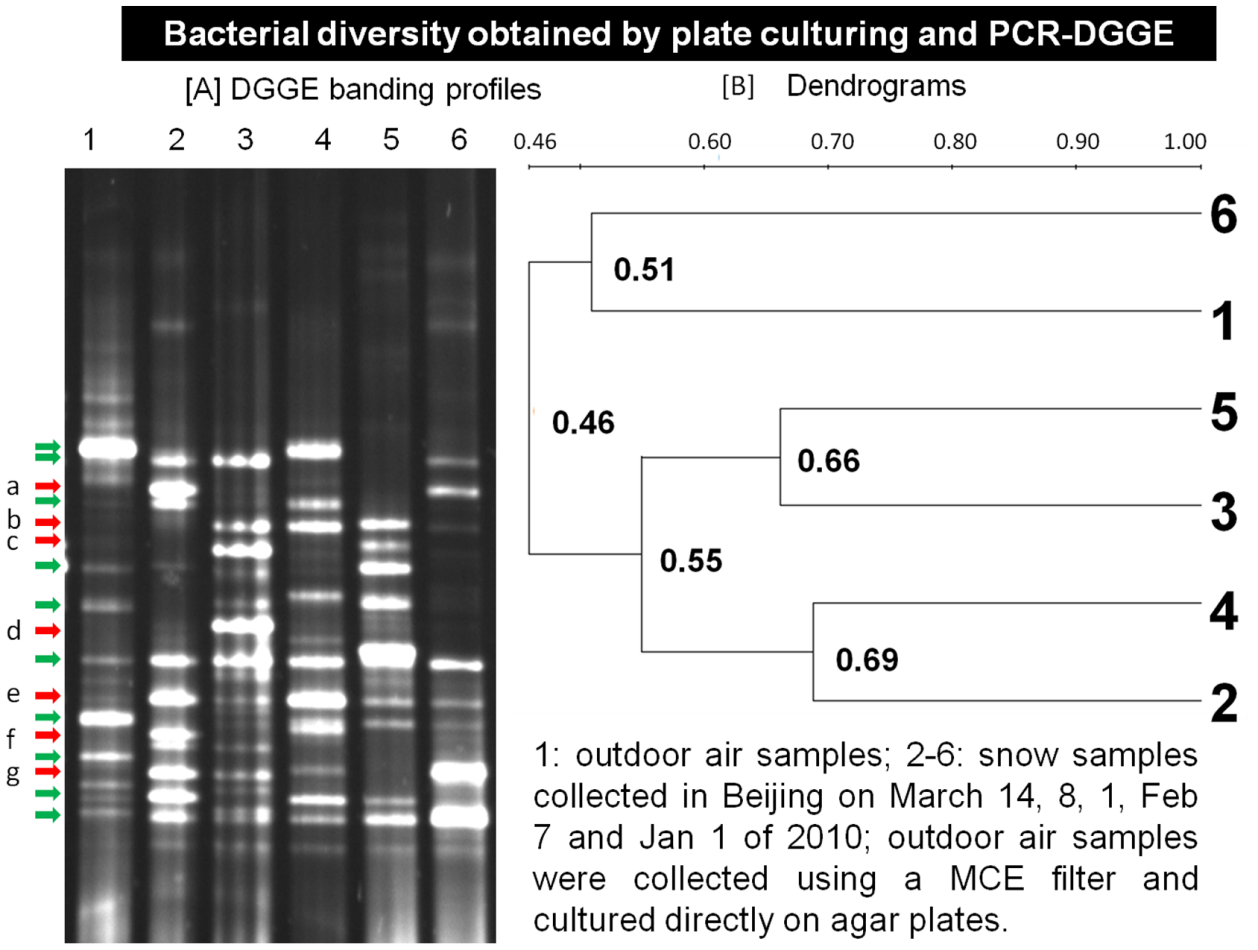
Bacterial diversity in snow samples. [A] PCR-DGGE banding profiles of five different snow samples and an outdoor air sample (540 L) collected in January-March, 2010, Beijing; [B] Dendrograms obtained from the DGGE profiles shown in [A]; the analysis of bacterial diversity in snow samples was based on the mixture of five independent snow samples collected from five locations on each individual date for each snow occurrence.

PCR-DGGE analysis also showed distinct differences in bacterial diversity among the snow samples from different occurrences as well as those in outdoor air samples. Fig. 2 shows the culturable bacterial diversities and the corresponding dendrograms analysis of five snow samples collected on different occurrences and outdoor air samples collected in Beijing during January-March 2010. As observed in Fig. 2 [A], there were distinct differences between the banding profiles of the outdoor air samples and those of the snow samples collected on different dates. Apparently, there were six bandings (a, b, c, d, e, f, g) marked by red arrows lacking in the lane of the outdoor air samples compared to those found in the snow samples. In addition, different banding profiles were also observed among the snow samples collected. For the snow samples, those collected on March 1 and March 14, 2010 exhibited more visible bandings compared to other snow samples. Dendrograms tree analysis shown in Fig. 2 [B] indicated that the snow sample collected on March 1 and March 14 had the highest similarity (∼70%), corresponding well to the banding profiles shown in Fig. 2 [A]. The outdoor air sample had lowest diversity similarity (∼50%) with the snow samples from five snow occurrences as observed in Fig. 2[B]. The diversity similarity among the snow samples from five snow occurrences was found close to 55%.

**Figure 3:**
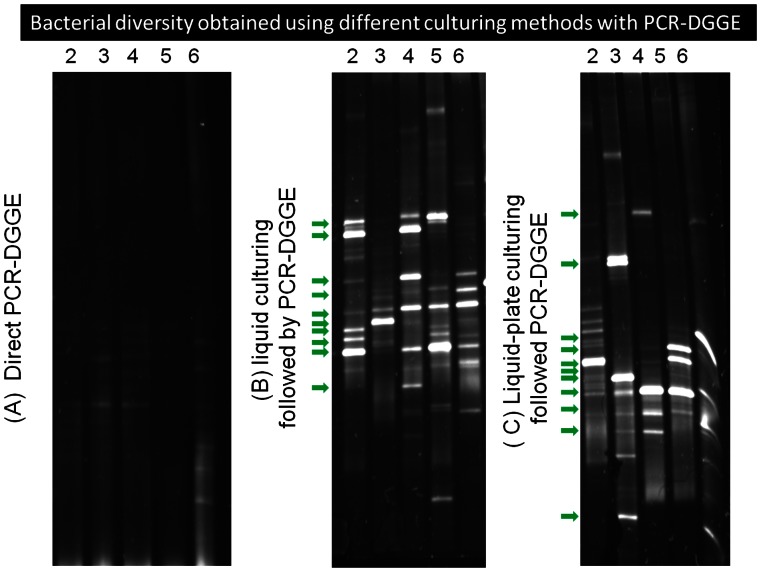
PCR-DGGE profiles(A, B, C) of the bacterial diversity of five different snow samples and an outdoor sample collected on different dates of the year 2010 in Beijing. (A) direct PCR-DGGE; (B) liquid culturing followed by PCR-DGGE, (C) liquid-plate culturing followed by PCR-DGGE; 2–6: snow samples collected in Beijing from five different occurrences that happened on March 14, 8, 1, Feb 7 and January 1 of 2010; the analysis of bacterial diversity in snow samples was based on the mixture of five independent snow samples collected from locations on each individual date; DI water was used as negative control for PCR-DGGE analysis.

**Figure 4:**
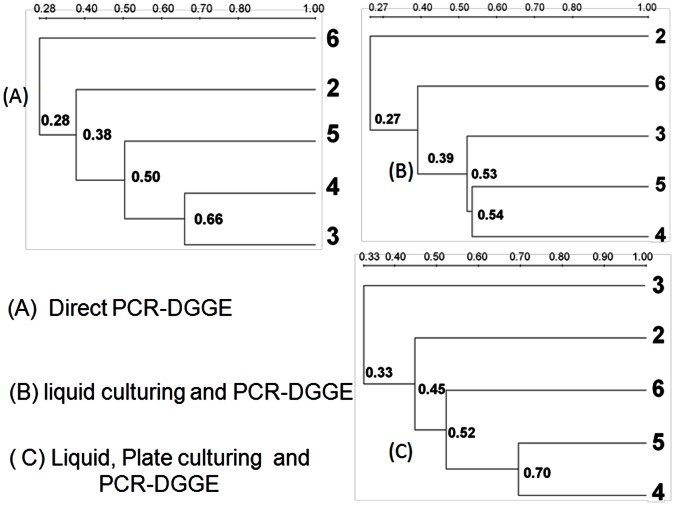
Dendrograms obtained from the DGGE profiles of different snow samples processed as described in Fig. **3.** Direct PCR-DGGE, liquid culturing followed by PCR-DGGE, and liquid-plate culturing followed by PCR-DGGE; 2–6: snow samples collected in Beijing from five different snow occurrences that happened on March 14, 8, 1, Feb 7 and January 1 of 2010.

In our work, different culturing methods for the bacterial diversity analysis were also investigated in addition to the agar plate culturing followed by PCR-DGGE. Fig. 3 shows the results obtained using three additional methods: direct PCR-DGGE, liquid culturing followed by PCR-DGGE, and liquid-plate culturing followed by PCR-DGGE. As observed in Fig. 3, the direct PCR-DGGE method resulted in poorest quality of DGGE banding profiles for all snow samples as a result of PCR inhibitions, which was likely due to the complex environmental matrix in the snow samples, while the liquid culturing followed by PCR-DGGE performed best among these three methods. Similar to the results obtained using agar plate culturing shown in Fig. 2, there were distinct differences observed in the bacterial diversities of the snow samples collected. Dendrograms tree analysis shown in Fig. 4 indicated that the diversity similarity was as low as 30% between some snow samples collected and the outdoor air samples. As observed in Fig. 2 and Fig. 3, different methods produced different banding profiles for the snow samples. Among the culturing methods investigated, the plate culturing followed by PCR-DGGE method produced the best banding profiles. For liquid culturing type methods, there might be fast bacteria growers in the snow samples, which could overgrow and suppress others.

**Figure 5:**
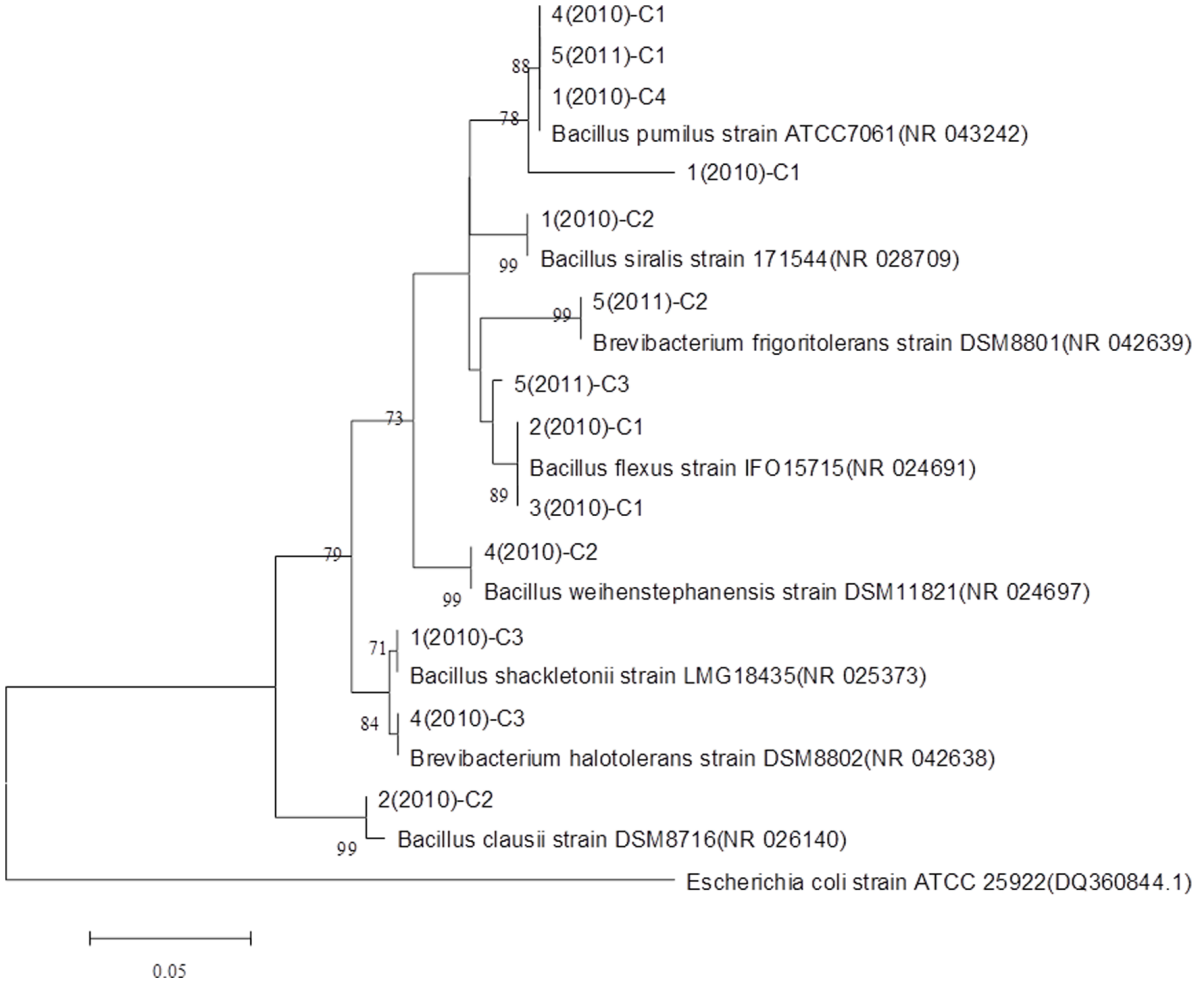
Neighbour-joining phylogenetic tree showing the relationship of representative sequences of OTUs in all snow samples and reference sequences in GenBank. It was constructed based on analysis of 16 S rRNA gene sequences of bacteria clone libraries from snow samples. Clone names from 1 (2010) to 4 (2010) represent samples collected on January 1, March 1, 8, 14, Feb 7 of 2010. Clone name 5 (2011) represents snow samples collected on Feb 27 of 2011. The capital “C” in the clone name means Clone. An *Escherichia coli* strain ATCC 25922 (dq 360844.1) was used as the outgroup.

Here, a V3 region of 16 S rRNA gene clone library was constructed from four snow samples collected in 2010 and one snow sample collected in 2011 to characterize the diversity of the snow bacterial communities and to identify the dominant taxonomic groups. As shown in Fig. 5, all snow samples were dominated by the representatives of genus *Bacillus*. On one hand, this finding might due to the liquid culturing method used to retrieve the bacteria of snow samples. Spore-forming organisms, such as *Bacillus* species and other Gram-positives, tend to dominate culture-dependent surveys of airborne microbial diversity [18]. On the other hand, *Bacillus* species show high resistance to environmental stresses, including UV light exposure, desiccation, and the presence of oxidizers [50]. In previous studies, *Bacillus* species had been collected from spacecraft and facility surfaces, and *Bacillus pumilus* was found to be the second most dominant species among the aerobic spore-forming bacteria [51], which was also found in snow samples. Bacterial strain closely related to *Bacillus flexus* was also isolated from surface snow samples collected from Antarctica [52]. Our results here imply that *Bacillus* species could serve as an efficient ice nucleator.

In a recent study, it was also shown that the bacterial communities found in the air appear to be distinctly different from the bacterial communities found in freshly fallen snow samples [49]. Similar to our work, it was shown that the certain bacterial species were found in two snow samples, but absent from the outdoor air samples collected. In their study, they have also shown that the diversity and composition of the airborne microbial communities including bacteria and fungi were also relatively stable over a two-week time period. To our best knowledge, the study by Bowers and co-workers (2009) was the first study to report the microbial diversity differences between air and snow samples [49]. However, in their study, they only collected two snow samples in a single snow precipitation. Here, we used five different snow samples collected from five snow precipitations that happened during January-March 2010. The differences in bacterial diversity found in five different snow samples as well as those in outdoor air samples indicate that microbial structure in the snow samples is dynamic and could be a footprint of a regional source compared to those in outdoor air sample.

### Fungal Diversity in the Snow Samples

In this study, the dominant fungal species in the snow samples and the outdoor air sample were also analyzed based on the morphology using the microscopy methods. Table 1 shows the dominant fungal species found in both snow and outdoor air samples by the plate culturing at 26°C. Among the fungal species identified as shown in Table 1, representatives of genera *Cladosporium*, *Penicillium*, *Aspergillus*, *Alternaria* were found abundant in most samples studied. For the outdoor air samples, *Alternaria*, *Aspergillus*, *Penicillium*, *Cladosporium*, *Stemphylium* and *Phyllosticta* species found to dominate the community. Likewise, we also detected *Cladosporium*, *Penicillium*, *Aspergillus*, *Alternaria* species in the snow samples collected. However, it should be noted that there were some species belonging to *Entomophthorales* (insects pathogen), *Saccharomyces cerevisiae* (human opportunistic pathogen [53], [54]), *Pseudocercospora* (plant pathogen), *Blastocladiales*, and *Leptomitales* found to be abundant in the snow samples, but not detected in the outdoor air sample. As indicated in Table 1, some of these fungal species in the snow samples are plant and insect pathogens, and they could have adverse effects on the local microbial ecology and agriculture when precipitated via snowfall to the local environment.

**Table 1:** Dominant fungal species (highest number of colonies obtained per unit of volume) in snow and outdoor air samples collected in Beijing from January to March, 2010.

Different snow samples	Dominant fungal species identified using microscopic methods
1-Jan	*Entomophthorales* (insects pathogens), *Cladosporium*, *Penicillium*, *Aspergillus*, *Alternaria*
1-Mar	*Aspergillus*, *Saccharomyces cerevisiae* (budding yeast), *Cladosporium*
8-Mar	*Pseudocercospora* (plant pathogens), *Cladosporium, Zoopagales* (parasites or predators of microscopic animals), *Alternaria*
14-Mar	*Blastocladiales*, *Cladosporium*, *Leptomitales*, *Alternaria*
Outdoor air sample	*Alternaria*, *Aspergillus*, *Penicillium*, *Cladosporium*, *Stemphylium* (plant pathogen), *Phyllosticta* (plant pathogen)

In a recent study, they also found fungal species in the snow samples collected [49], however the community was dominated by *Dothideomycetes* and *Eurotiomycetes* of fungi not by common species such as *Alternaria*, *Cladosporium*, and *Penicillium* spp. They attributed this to the culturing bias. For the outdoor air sample, they found 1∶1 ratio of bacterial and fungal sequences. In another study, it was demonstrated that species diversity of fungi in the snow was much broader than in aerosols [43]. Similar to our work, they have also found *Alternaria* and *Cladosporium* genera in addition to others such as *Botrytis* spp. and *Ulocladium* spp. in their snow samples. Laboratory studies suggest that fungi can also act as the ice nuleator, initiating the snow precipitation [45], [46]. The global transport of fungal species have been widely reported [18], [28], [29]. During the snow precipitation, such transported species could descend to the ground, thus causing impacts on the local microbial ecology and the agriculture health. Our data add to the body of the evidences for such possibilities.

### Conductance and Total Organic Carbon (TOC) in the Snow Samples

**Figure 6:**
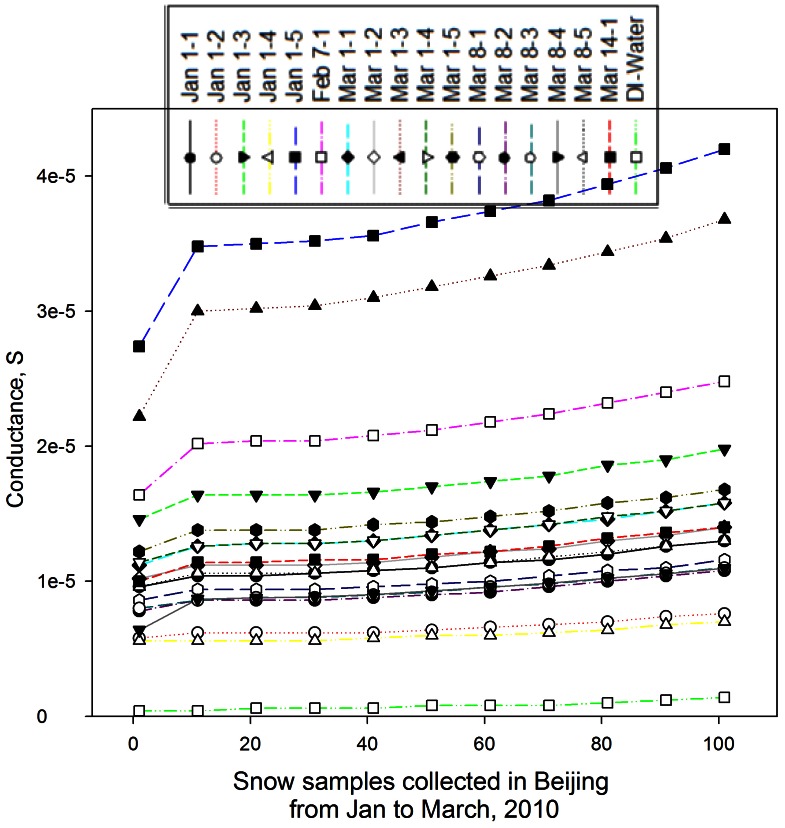
Conductance levels of the five different melted snow samples collected in January-March, 2010 in Beijing measured at different modulation frequencies of 1 kHz to 100 kHz by a lock-in amplifier operated at a modulation amplitude of 50 mV. A LabView program was develoepd to automatically modify the output modulation frequency; data points represent averages of 500 measurements (1 measurement per 300 millisecond); the arrow indicates the conductance transition at the frequency of 10 kHz.

**Figure 7:**
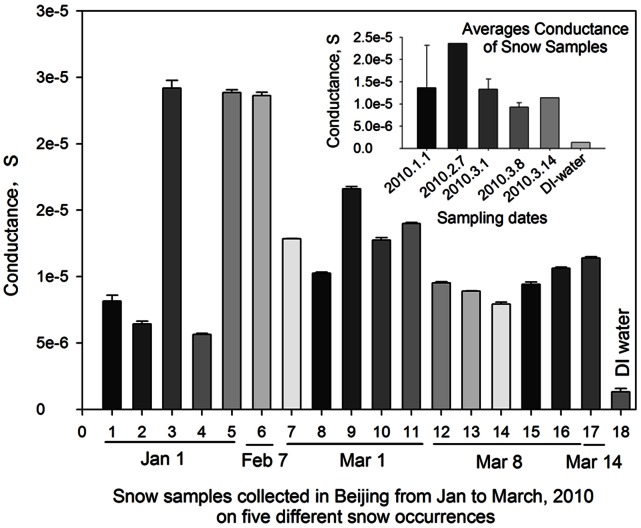
Average conductance levles measured for five different snow samples collected on different dates and DI water (Millipore) using a lock-in amplifier at a modulation frequency of 100 kHz. Inset figure represents the conductance averages of the mixture of snow samples collected from five different locations on each individual dates.

Bacteria and fungi are important contributor to both conductance and total organic carbon [16], [55], [56], [57]. In this work, we also observed significant differences in the conductance levels of the melted snow samples and the outdoor air samples (after DI water extraction). Fig. 6 shows the results of the conductance levels for melted snow samples and DI water at a modulation frequency from 1 to 100 kHz. As observed from Fig. 6, the conductance levels of the snow samples from five different snow occurrences increased with increasing modulation frequency. At the frequency of higher than 10 kHz, the increase of the conductance level was slowed down slightly. At 100 kHz, the conductance levels remained relatively stable for most snow samples. As shown in Fig. 6, the conductance levels of the individual snow samples were substantially higher than that of DI water. In a previous study, 100 kHz was used to measure the bacterial concentrations using the impedance based technology [56]. Accordingly, 100 kHz was used in this work to measure the conductance levels of the snow samples (the mixtures of five snow samples from five different locations collected for each snow occurrence on each individual date) as shown in Fig. 7. From Fig. 7, it was observed that the average conductance levels varied with snow samples, but substantially higher than that of DI water. Compared to bacterial concentrations in relevant snow samples shown in Fig. 1, no exact correlation between the conductance and bacterial concentration can be drawn (R^2^<0.1). In our another study, we have shown that the bacterial suspension condutance is an exponential function of bacterial concentration for pure bacterial cultures [57]. In a recent study, it was shown that the conductivity of surface snow varied with seasons, and Ca^2+^ was found the key ion in determining the conductivity of the surface [33]. Here, the conductance levels for the snow samples might be a result of both baterial abundance and other dissolved chemical ions.

**Figure 8:**
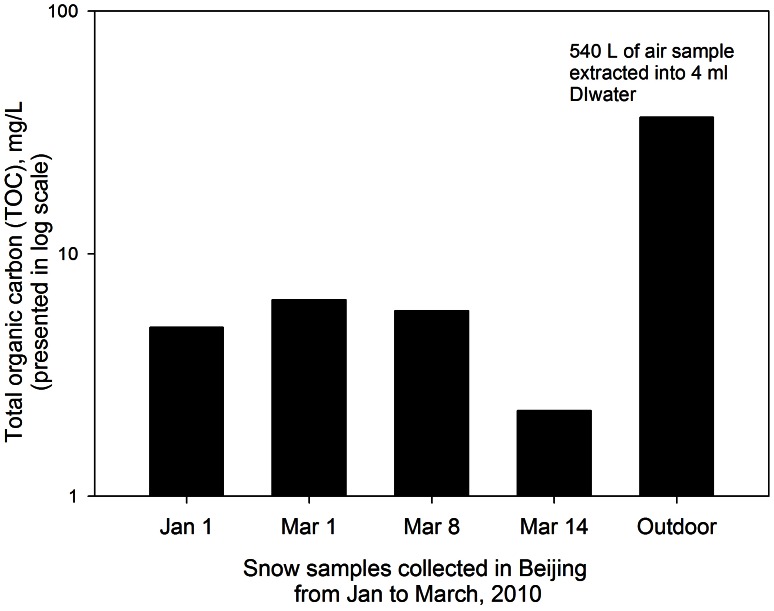
The total organic carbon (TOC) concentrations (mg/L) in snow samples and an outdoor air sample collected in January-March, 2010 in Beijing. Data points represent averages of the mixture of snow samples collected from five different locations; outdoor air samples were collected using a MCE filter.

It was shown that bacterial and fungal species contribute to the total carbon in the atmosphere [16], [55]. Here, the total oragnic carbon (TOC) in the snow samples and the outdoor air sample were also measured and presented in Fig. 8. In this work, TOC levels with similar magnitudes were found among different snow samples, however for the outdoor air samples (540 L of air extracted into 4 ml DI water) the TOC levels were shown to be substantially higher, ranging from 6 to 16 times of those of the snow samples. However, it is difficult to quantitatively assess the comparison because of different collection methods for the snow samples and the outdoor air. According to the conversion factor of 17 fg carbon per bacterium [42], culturable bacteria in the snow samples collected in this work were calculated to account for an average of 3.38%(±1.96%) of TOC, while those in the outdoor air samples accounted for about 0.01%. Therefore, outdoor air sample might have had larger TOC contribution from other sources rather than the bacteria than the snow samples. In a previous study, it was found that the most abundant group of organic compounds in the aerosol samples were phthalic acid esters with diisobutylphthalate as the main substance, which was considered to originate from local emisisons [58]. In their study, they also found that diisobutylphthalate was the dominant substance in snow samples. It was shown in a previous study that total bacteria in cloud water accounted for about 1.5% of TOC [16]. In this work, only culturable bacteria were used, thus the actual contributions could be higher if those non-living ones are also included.

## Discussion

Increasing evidences suggest that the atmosphere is a habitat for bioaerosols where these biological particles may affect the precipitation cycle and/or their own precipitation during the atmospheric transport [1]. As discussed, evidences are there to support that the biological agents could be transported globally. It was shown that increased levels of fungi were found in Taiwan during the dust events that occurred in China [24]. In another study, it was suggested that dust storms could have been a possible source of foot-and-mouth disease virus outbreaks that occurred in Korea and Japan, and some of these outbreaks were observed following the Gobi/Takla Makan dust events [20], [22], [23]. In a recent review article, it was concluded that the biological agents including bacteria, fungi and virus could survive the long-range transport [27]. It was also indicated that billions of tons of desert dust could move through the atmosphere each year [27]. The particles during such dust storms possibly prevent bacteria and fungi from the inactivating effects of UV exposure, accordingly global transport of dust will have more far-reaching effects in addition to impaired visibility [59]. These transported pollutants could further settle into the local environments via snow and rain precipitations. In this work, we have observed distinct differences in bacterial and fungal diversities obtained for the snow samples collected from five snow occurrences and the outdoor air sample. Those species, foreign to the local environment, could have been transported together with mineral dusts from remote regions. Among the species transported, in addition to human disease causing agents some of the ice nuleators such as *Pseudomonas syringae*, are plant pathogens [19], which could impose an agriculture harm when precipitated into the local environment via snowfalls. In this work, we have found human, plant and insect pathogens in the snow samples as listed in Table 1, and these species could present environmental risks.

For the snow samples collected in this study, they might have captured biological particles during their transport to the ground. However, this process would not affect the conclusion from this study. In this work, all snow samples went through the same process, and distinct bacterial diversities were also found in the snow samples. In this study, 16 S rRNA gene-targeted PCR-DGGE was performed on both snow samples and the outdoor air sample. The results obtained here could be negatively impacted by certain DGGE limitations such as the intraspecies operon heterogeneities. In addition, use of microscopy methods, despite of wide use, for fungal identification would have some limitations on the morphology identification and corresponding comparison. Therefore, the results obtained for fungal diversity in this study might have its method limitations. In this study, MCE filter was used for the outdoor air sample analysis. In our recent work, we have found out that highest bacterial diversity was obtained when MCE filter with air samples was directly placed on agar plates for culturing compared to other available bioaerosol sampling methods [40]. Nonetheless, filter sampling could possibly result in loss of certain species, and thus such limitation should be taken into account for the results obtained here.

### Conclusions

Bioaerosols are increasingly being recongonized as a player in the cloud formation, atmospheric chemical process, and climate change. In this study, the bacterial and fungal abundance and diversities in the snow samples collected in Beijing were investigated. The results have shown that there were not only high levels of bacterial concentrations, but also distinct differences in both bacterial and fungal diversities including human, insect and plant pathogens from different snow samples. These results suggested that the microbial structure in the cloud could change dynamically over the time, likely due to a microbial flux transported from other places. In addition, similar to a previous sudy [49], there were strikingly different bacterial diversity diffferences observed between the snow samples and the outdoor air samples. This finding supports a possible regional and/or global transport of microbial agents through the cloud movement. In addition, our clone libratory also indicates that *Bacillus* species could possibly serve as an efficient ice nucleator. The precipiation of these foreign biological agents via snowfall or rainfall could have non-negligible impacts on local climate, human health and agriculure security. In addition, snow precipitation could be one of major contributors to the TOC in local environments. In this study, small sample size, i.e., only five snow occurrences, despite of unusual high frequency for Beijing within a year, could have possible limitations on the conclusion generated.
